# Microwave Assisted Organic Synthesis (MAOS) of Small Molecules as Potential HIV-1 Integrase Inhibitors

**DOI:** 10.3390/molecules16086858

**Published:** 2011-08-11

**Authors:** Stefania Ferro, Sara De Grazia, Laura De Luca, Rosaria Gitto, Caterina Elisa Faliti, Zeger Debyzer, Alba Chimirri

**Affiliations:** 1 Department of Medicinal Chemistry, University of Messina, Viale Annunziata, I-98168 Messina, Italy; Email: sdegrazia@pharma.unime.it (S.D.G.); ldeluca@unime.it (L.D.L.); rgitto@unime.it (R.G.); katia_chem@tiscali.it (C.E.F.); achimirri@unime.it (A.C.); 2 Molecular Medicine, Katholieke Universiteit Leuven and IRC KULAK, Kapucijnenvoer 33, B-3000 Leuven, Flanders, Belgium; Email: zeger.debyser@med.kuleuven.be (Z.D.)

**Keywords:** AIDS, integrase inhibitors, small molecules, MAOS

## Abstract

Integrase (IN) represents a clinically validated target for the development of antivirals against human immunodeficiency virus (HIV). In recent years our research group has been engaged in the stucture-function study of this enzyme and in the development of some three-dimensional pharmacophore models which have led to the identification of a large series of potent HIV-1 integrase strand-transfer inhibitors (INSTIs) bearing an indole core. To gain a better understanding of the structure-activity relationships (SARs), herein we report the design and microwave-assisted synthesis of a novel series of 1-*H*-benzylindole derivatives.

## 1. Introduction

Human Immunodeficiency Virus 1 (HIV-1) is the etiological agent of Acquired Immune Deficiency Syndrome (AIDS) which has caused a large number of deaths for over 20 years. During this period, many efforts have been made to discover antiretroviral drugs that are able to block HIV-1 replication by targeting different but crucial steps of its life cycle.

To date, the U.S. Food and Drug Administration (FDA) has approved drugs that are often used in combination regimens in the well-known Highly Active Antiretroviral Therapy (HAART). These can be divided into seven groups: Nucleoside reverse transcriptase inhibitors (NRTIs), nucleotide reverse transcriptase inhibitors (NtRTIs), non-nucleoside reverse transcriptase inhibitors (NNRTIs), protease inhibitors (PIs), fusion inhibitors (FIs), co-receptor inhibitors (CRIs), and integrase inhibitors (INIs) [1]. Although this current therapy has been successful in substantially decreasing in morbidity and mortality rates, the use of this arsenal of drugs is limited by toxicity, adverse effects, drug resistance, and more worryingly by the fact that some newly HIV-infected patients carry viruses that are already resistant to currently approved AIDS treatments [2,3]. These issues make it apparent that the discovery and the development of new more potent and less toxic anti-HIV agents are urgently required.

Considering that the stability of the infection depends critically on the insertion of viral DNA into the host genome, the integration process mediated by HIV-1 Integrase (IN) has become an attractive and a promising target. HIV-1 IN is a 32 kDa protein that belongs to the superfamily of polynucleotidyl transferases, comprising three structural domains: (1) the amino-terminal domain (NTD); (2) a catalytic core domain (CCD); and (3) the carboxyl-terminal domain (CTD) [4,5].

The insertion of a double-stranded DNA copy into the chromosomes of an infected cell comprises a multistep process that involves two separate reactions: 3’-processing (3’-P) and strand-transfer (ST). In the first step, IN leads to the formation of new 3’-CA-OH while the ST step is a trans-esterification reaction involving the direct nucleophilic attack of the 3’-hydroxy group of the two newly processed viral 3’-DNA ends on the phosphodiester backbone of the host target DNA. Finally, the two overhanging 5’-end nucleotides are cleaved, and the target DNA is then integrated into cellular DNA. No energy source (e.g., ATP) is needed for the integration reaction, and only divalent cations are required for catalytic activity. In particular, the two metal cofactors of HIV-IN are both thought to be Mg^2+^ ions under physiological conditions [6,7,8,9,10].

**Figure 1:**
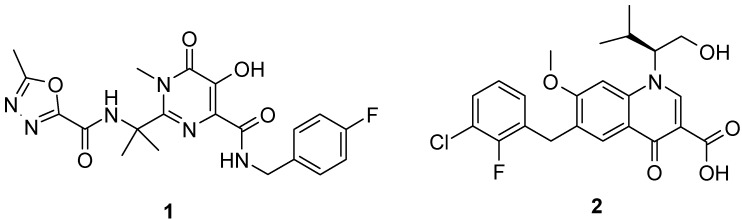
Chemical structures of INSTIs: Raltegravir (**1**) and elvitegravir (**2**).

To date, all of the small-molecules that have claimed potent antiviral activity as integrase strand-transfer inhibitors (INSTIs) contain a structural motif able to coordinate the two divalent metal ions in the active site of the enzyme. Although we now have a good collection of potential INSTIs, there is only one FDA-approved HIV-IN inhibitor, raltegravir (**1**, IC_50_ = 0.0068 μM), marketed as Isentress and one more, elvitegravir (**2**, IC_50_ = 0.002 μM) which is undergoing phase III clinical trials [11,12] (Figure 1).

**Figure 2:**
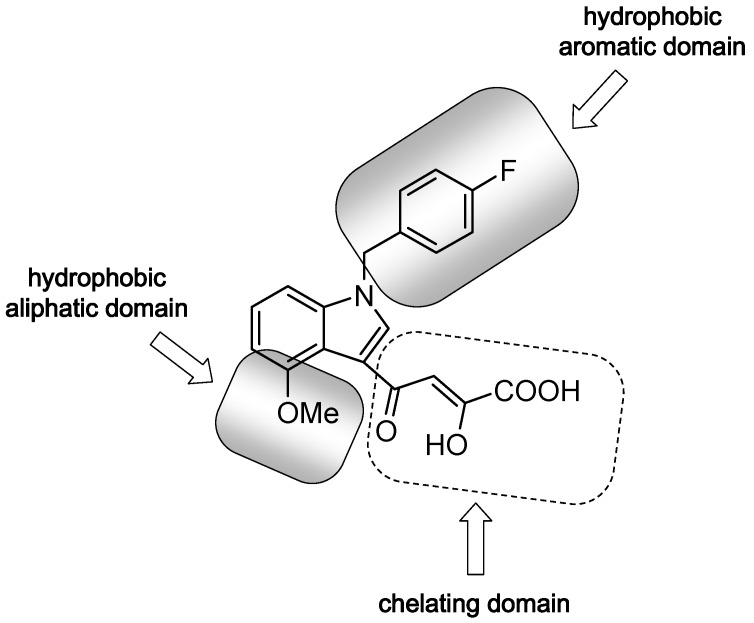
Chemical structure of CHI-1043 (**3**); Major structural domains are highlighted.

On these bases and taking into account the structural requirements necessary to form a ligand-Mg^2+^-IN complex [13,14], in recent years our research group has been engaged in the structure-function study of HIV-IN and in the development of new inhibitors able to selectively inhibit the ST step of the integration. Previously, we reported ligand-based approaches that are useful to generate three-dimensional pharmacophore models which led to the identification of a large series of indole DKA analogs as novel and potent INST inhibitors [15,16,17,18,19,20]. In particular, we discovered the potent derivative **3**, CHI-1043 (Figure 2), that displayed the best anti-HIV activity, thus holding promise for further development [20,21].

We herein report the rational design and synthesis of new methyl-benzylindoles CHI-1043 analogues with the aim of expanding the SARs (structure-activity relationships) of this class of compounds and of improving both their activity and selective index (SI). The use of high-speed technology MAOS (Microwave-Assisted-Organic-Synthesis) provided the following advantages: (i) controlled heating conditions; (ii) reaction time and solvent reduction; (iii) faster exploration of SARs.

## 2. Results and Discussion

Our ligand- and structure-based drug design studies highlighted the importance of the hydrophobic feature of a *p*-fluorobenzyl ring on CHI-1043 derivatives. Taking into account these findings in which we previously reported a large series of halogen-substituted derivatives [17,18], we went on to plan the synthesis of some new 1*H*-benzylindoles bearing one or more methyl groups on the different positions of the benzyl moiety, with the aim of gaining a better understanding of the SAR of these series of compounds. In this work we focus on the use of an efficient and practical method for the rapid preparation of 1-*H*-benzyl indole derivatives as potential INSTIs under microwave conditions.

**Scheme 1:**
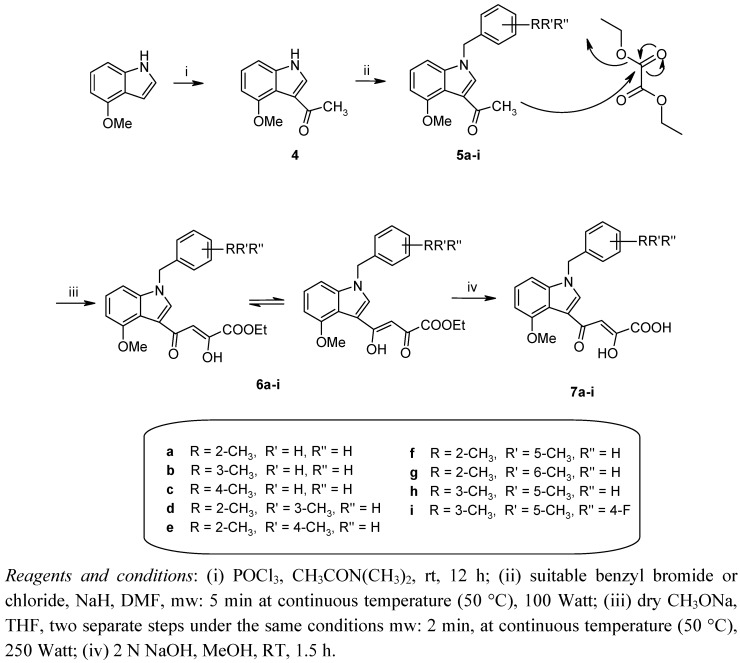
Schematic representation for the synthesis of compounds **7a–i**.

This innovative and eco-friendly technology has the potential to influence medicinal chemistry work in the main phases of the drug discovery process such as the generation of chemical libraries of “small molecules” or lead optimization. Unlike conventional conditions in which heat is transferred by conductance, microwave irradiation produces efficient internal heating by the direct coupling of microwave energy with the polar molecules present in the reaction mixture. Simple, quick, and efficient MAOS reactions frequently occur much faster than under conventional heating, often providing higher yields of the desired products which can be more easily and rapidly purified [22]. The chemical synthesis of the designed compounds was carried out as depicted in Scheme1.

Commercially available 4-methoxy-1*H*-indole was first 3-acetylated by a Vilsmeier Haack reaction, in which the formylating agent was generated *in situ* from dimethylacetamide and phosphorus oxychloride. Reaction of the obtained intermediate **4** with the suitable benzylbromide or chloride gave the corresponding *N*-alkyl derivatives **5a–i**. This step of the synthetic pathway was carried out by the addition of sodium hydride in dimethylformamide at 50 °C for 5 min under 100 W by the application of Microwave-Assisted-Organic-Synthesis (MAOS).

To synthesize diketoester derivatives **6a–i** we employed the following two-step sequence under the same conditions: 50 °C for 2 min under 250 W of microwave irradiation. Benzyl derivatives **5a–i** reacted with diethyl oxalate providing a mixture of tautomers which without separation were converted into the corresponding diketo acids **7a–i** by hydrolysis in basic medium. All the desired products were fully characterized by elemental analysis, ^1^H-NMR, and ^13^C-NMR.

In order to determine the susceptibility of the HIV-1 integrase enzyme towards all of the synthesized diketo esters and diketo acids, they were tested by enzyme-linked immunosorbent assays. Their inhibitory effect on the HIV-induced cytopathic effect (CPE) in human lymphocyte MT-4 cell cultures was also determined using the MT-4/MTT-assay. The results obtained were compared with those of our “lead compound” CHI-1043 (**3**).

The analysis of the enzymatic inhibition confirmed our previously reported results, showing also in this case major activity for the diketo acids 7a–i in comparison to the corresponding diketo esters 6a–i (1.08 < µM < 55.85 *vs.* 17.02 < µM < 79.65 on ST step). The mono-methyl substitution on the benzyl moiety seems to positively influence the IN binding (compounds 7a–c), of these the *ortho*- and *meta*-methyl substituted compounds 7a and 7b proved to have the best IC_50_ values: 1.07 μM and 1.36 μM, respectively (Table 1).

**Table 1:** Inhibition of HIV-1 integrase enzymatic activity.

Compound	IN enzymatic activity (IC_50_-µM)	
	^a^ Overall	^b^ ST
**7a**	1.74 ± 1.32	1.07 ± 0.76
**7b**	0.42 ± 0.29	1.36 ± 0.64
**7c**	5.76 ± 1.82	8.84 ± 5.60
**7d**	2.61 ± 1.17	6.79 ± 0.64
**7e **	51.76 ± 33.81	55.85 ± 43.83
**7f**	3.83 ± 2.21	9.47 ± 1.06
**7g**	7.34 ± 1.32	23.54 ± 17.78
**7h**	7.69 ± 5.59	5.49 ± 2.67
**7i **	2.17 ± 1.65	5.24 ± 1.34
**CHI-1043**	0.08 ± 0.003	0.14 ± 0.11

^a^ Concentration required to inhibit the *in vitro* overall integrase activity by 50%; ^b^ Concentration required to inhibit the *in vitro* strand transfer step by 50%.

By comparison of these data with those of our 4-fluorosubstituted lead compound CHI-1043 we hypothesized that the substituent in the *para* position of the benzyl moiety would positively influence the activity due not to its lipophilicity (*i.e.*, Me), but more probably due to its electronic effect and/or ability to form an additional hydrogen bond.

Association of *2*-substitution with another methyl group provided a gradual decrease of IN inhibition showing the most interesting result for derivative **7d** (IC_50_ = 6.79 μM), which is characterized by the presence of 2,3-dimethyl substitution on the benzyl moiety, and the worst for 2,4- and 2,6-dimethyl substitution (**7e** and **7g**, Table 1). **A** comparable IC_50_ value (5.49 μM) was obtained for derivative **7h**, bearing the 3,5-dimethyl substitution, while an additional fluorination at the 4-position (compound **7i**) led to only a marginal improvement in activity (IC_50_ = 5.24 μM).

When the synthesized compounds were tested against HIV-1 replication in cell cultures, the *2*- and *3*-methyl substituted derivatives showed good EC_50_ values: 1.74 μM (**7a**) and 2.14 μM (**7b**). Notably, compound **7a** displayed the best selectivity index (SI = 43), measured as a ratio of EC_50_ and CC_50_ values.

Considering the disubstituted derivatives, the results obtained for compound **7d** (EC_50_ = 1.74μM) further confirmed the positive effect of the methyl group when it occupies the 2- and 3- positions of the benzyl moiety.

## 3. Experimental

### 3.1. Chemistry

#### 3.1.1. General

All the microwave-assisted reactions were carried out in a CEM Focused Microwave Synthesis System, Model Discover, working at the power required for refluxing under atmospheric conditions. Melting points were determined on a Buchi Melting Point B-545 apparatus and are uncorrected. Elemental analyses (C, H, N) were carried out on a Carlo Erba Model 1106 Elemental Analyzer and the results were within ±0.4% of the theoretical values. Merck silica gel 60 F_254_ plates were used for analytical TLC; column chromatography was performed on Merck silica gel 60 (230–400 mesh), and Flash Chromatography (FC) on a Biotage SP_1_ EXP. ^1^H-NMR spectra were recorded in CDCl_3_ with TMS as internal standard or DMSO-*d*_6_ on a Varian Gemini-300 spectrometer. Chemical shifts were expressed in δ (ppm) and coupling constants (*J*) in hertz (Hz). All the exchangeable protons were confirmed by addition of D_2_O. 3-Acetyl-4-methoxy-1*H*-indole (**4**) was synthesized following the previously reported procedure [18]. Briefly, phosphoryl chloride (0.92 mL, 10 mmol) was added to ice cold dimethylacetamide (2.79 mL, 30 mmol) under stirring and cooling in ice. 4-Methoxy-1*H*-indole 5 (147.18 mg, 1 mmol) was added and the reaction mixture was stirred at room temperature for 12 h, then poured over ice and basified with 4 N aqueous sodium hydroxide solution. The mixture was extracted with ethyl acetate and dried over Na_2_SO_4_. After removal of the solvent under reduced pressure, the residue was crystallized by treatment with diethyl ether and recrystallized from dichloromethane. mp 113–115 °C, yield 89%. ^1^H-NMR (δ) 2.70 (s, 3H, CH_3_), 3.97 (s, 3H, OCH_3_), 6.68–7.79 (m, 4H, ArH), 8.69 (br s, 1H, NH). Anal. Calcd for C_11_H_11_NO_2_: C, 69.83; H, 5.86; N, 7.40. Found: C, 69.62; H, 5.63; N, 7.51.

#### 3.1.2. General procedure for the synthesis of 3-acetyl-4-methoxy-1-benzyl-1*H*-indoles **5a–i**

Using the synthetic procedure previously reported by us [17,18], 3-acetyl-4-methoxy-1*H*-indole (189 mg, 1 mmol) was dissolved in DMF (1 mL) at 0 °C and dry sodium hydride (120 mg, 5 mmol) was added. After stirring for 2 min, the appropriate benzyl bromide (1.5 mmol) was added dropwise. The resulting solution was placed in a cylindrical quartz tube (diam. 2 cm), stirred and irradiated in a microwave oven at 100 W and at continuous temperature (50 °C) for 5 min. A saturated NaHCO_3_ solution was added. The reaction mixture was extracted with ethyl acetate (10 mL × 3) and dried over Na_2_SO_4_. After removal of the solvent under reduced pressure, the crude mixture was purified by flash chromatography using a mixture of cyclohexane/ethylacetate (6:4).

*3-Acetyl-1-(2-methylbenzyl)-4-methoxy-1H-indole* (**5a**). Yield: 64%; mp: 138–140 °C. ^1^H-NMR (CDCl_3_): δ = 2.29 (s, 3H, CH_3_), 2.66 (s, 3H, CH_3_), 3.98 (s, 3H, OCH_3_), 5.24 (s, 2H, CH_2_), 6.61 (d, 1H, *J* = 7.9, ArH), 6.83 (d, 1H, *J* = 7.4, ArH), 6.95 (d, 1H, *J* = 8.2, ArH), 7.12–7.25 (m, 3H, ArH), 7.56 ppm (s, 1H, ArH); Anal. calcd for C_19_H_19_NO_2_: C 77.79, H 6.53, N 4.77, found: C 77.83, H 6.49, N 4.73.

*3-Acetyl-1-(3-methylbenzyl)-4-methoxy-1H-indole* (**5b**). Yield: 73%; mp: 117–119 °C. ^1^H-NMR (CDCl_3_): δ = 2.31 (s, 3H, CH_3_), 2.70 (s, 3H, CH_3_), 3.98 (s, 3H, OCH_3_), 5.26 (s, 2H, CH_2_), 6.70 (d, *J* = 7.9, 1H, ArH), 6.93–6.96 (m, 3H, ArH), 7.12–7.25 (m, 3H, ArH), 7.73 ppm (s, 1H, ArH); Anal. calcd for C_19_H_19_NO_2_: C 77.79, H 6.53, N 4.77, found: C 77.62, H 6.38, N 4.62.

*3-Acetyl-1-(4-methylbenzyl)-4-methoxy-1H-indole* (**5c**). Yield: 61%; mp: 100–102 °C. ^1^H-NMR (CDCl_3_): δ = 2.31 (s, 3H, CH_3_), 2.67 (s, 3H, CH_3_), 3.97 (s, 3H, OCH_3_), 5.25 (s, 2H, CH_2_), 6.68 (d, *J* = 7.7, 1H, ArH), 6.95–7.20 (m, 6H, ArH), 7.71 ppm (s, 1H, ArH); Anal. calcd for C_19_H_19_NO_2_: C 77.79, H 6.53, N 4.77, found: 77.56, H 6.39, N 4.93.

*3-Acetyl-1-(2,3-dimethylbenzyl)-4-methoxy-1H-indole* (**5d**). Yield: 99%; mp: 145–147 °C. ^1^H-NMR (CDCl_3_): δ = 2.16 (s, 3H, CH_3_), 2.32 (s, 3H, CH_3_), 2.66 (s, 3H, CH_3_), 3.98 (s, 3H, OCH_3_), 5.25 (s, 2H, CH_2_), 6.70–6.74 (m, 2H, ArH), 6.96–7.14 (m, 2H, ArH), 7.17–7.26 (m, 2H, ArH), 7.52 (s, 1H, ArH); Anal. calcd for C_20_H_21_NO_2_: C 78.15, H 6.89, N 4.56, found: C 78.23, H 6.71, N 4.38.

*3-Acetyl-1-(2,4-dimethylbenzyl)-4-methoxy-1H-indole* (**5e**). Yield: 60%; mp: 114–116 °C. ^1^H-NMR (CDCl_3_): δ = 2.23 (s, 3H, CH_3_), 2.32 (s, 3H, CH_3_), 2.66 (s, 3H, CH_3_), 3.99 (s, 3H, OCH_3_), 5.20 (s, 2H, CH_2_), 6.71 (d, *J* = 7.9, 1H, ArH), 6.78 (d, *J* = 7.9, 1H, ArH), 6.94–6.98 (m, 2H, ArH), 7.05 (s, 1H, ArH), 7.18–7.26 (m, 1H, ArH), 7.54 (s, 1H, ArH); Anal. calcd for C_20_H_21_NO_2_: C 78.15, H 6.89, N 4.56, found: C 78.41, H 6.72, N 4.71.

*3-Acetyl-1-(2,5-dimethylbenzyl)-4-methoxy-1H-indole* (**5f**). Yield: 63%; mp: 130–132 °C. ^1^H-NMR (CDCl_3_): δ = 2.23 (s, 6H, CH_3_), 2.67 (s, 3H, CH_3_), 3.99 (s, 3H, OCH_3_), 5.19 (s, 2H, CH_2_), 6.71–6.73 (m, 2H, ArH), 6.98–7.21 (m, 4H, ArH), 7.54 (s, 1H, ArH); Anal. calcd for C_20_H_21_NO_2_: C 78.15, H 6.89, N 4.56, found: C 78.39, H 7.01, N 4.21.

3*-Acetyl-1-(2,6-dimethylbenzyl)-4-methoxy-1H-indole* (**5g**). Yield: 78%; mp: 140–142 °C. ^1^H-NMR (CDCl_3_): δ = 2.24 (s, 6H, CH_3_), 2.60 (s, 3H, CH_3_), 3.98 (s, 3H, OCH_3_), 5.20 (s, 2H, CH_2_), 6.75 (d, *J* = 7.7, 1H, ArH), 7.12–7.32 (m, 6H, ArH); Anal. calcd for C_20_H_21_NO_2_: C 78.15, H 6.89, N 4.56, found: C 78.46, H 6.69, N 4.68.

*3-Acetyl-1-(3,5-dimethylbenzyl)-4-methoxy-1H-indole* (**5h**). Yield: 73%; mp: 110–112 °C. ^1^H-NMR (CDCl_3_): δ = 2.26 (s, 6H, CH_3_), 2.69 (s, 3H, CH_3_), 3.98 (s, 3H, OCH_3_), 5.20 (s, 2H, CH_2_), 6.69 (d, *J* = 7.9, 1H, ArH), 6.77 (s, 1H, ArH), 6.93–6.97 (m, 2H, ArH), 7.19 (t, *J* = 7.97, 1H, ArH), 7.72 (s, 1H, ArH); Anal. calcd for C_20_H_21_NO_2_: C 78.15, H 6.89, N 4.56, found: C 78.23, H 6.71, N 4.38.

*3-Acetyl-1-(4-fluoro-3,5-dimethylbenzyl)-4-methoxy-1H-indole* (**5i**). Yield: 61%; mp: 128–130 °C. ^1^H-NMR (CDCl_3_): δ = 2.20 (s, 6H, CH_3_), 2.69 (s, 3H, CH_3_), 3.97 (s, 3H, OCH_3_), 5.16 (s, 2H, CH_2_), 6.69 (d, *J* =7.9, 1H, ArH), 6.80 (d, *J* = 6.6, 2H, ArH), 6.93 (d, *J* = 8.2, 1H, ArH), 7.16–7.21 (m, 1H, ArH), 7.69 (s, 1H, ArH); Anal. calcd for C_20_H_20_FNO_2_: C 73.83, H 6.20, N 4.30, found: C 73.43, H 6.24, N 4.46.

#### 3.1.3. General procedure for the synthesis of 4-[1-benzyl-4-methoxy-1*H*-indol-3-yl]-2-hydroxy-4-oxobut-2-enoates **6a–i**

Adopting the synthetic procedure previously reported by us [17,18,20], a mixture of 3-acetyl-4-methoxy-1-benzyl-1*H*-indole (1 mmol), diethyl oxalate (219 mg, 1.5 mmol) and a catalytic amount of NaOCH_3_ was suspended in anhydrous THF (2 mL). The reaction mixture was placed in a cylindrical quartz tube (diam. 2 cm), stirred and irradiated at continuous temperature in a microwave oven for two successive time intervals under the same conditions (250 Watt, 2 min, 50 °C). The solvent was concentrated under reduced pressure and collected yellow solid was crystallized from ethanol and diethyl ether (1:4).

*4-[1-(2-Methylbenzyl)-4-methoxy-1H-indol-3-yl]-2-hydroxy-4-oxobut-2-enoate* (**6a**). Yield: 96%; mp: 167 °C dec. ^1^H-NMR (DMSO-*d*_6_): δ = 1.22 (t, *J* = 7.1, 3H, CH_3_), 2.24 (s, 3H, CH_3_), 3.84 (s, 3H, OCH_3_), 4.09 (q, *J* = 7.1, 2H, CH_2_), 5.36 (s, 2H, CH_2_), 6.59–7.44 (m, 9H ArH and CH); Anal. calcd for C_23_H_23_NO_5_: C 70.21, H 5.89, N 3.56, found C 70.32, H 5.76, N 3.69.

*4-[1-(3-Methylbenzyl)-4-methoxy-1H-indol-3-yl]-2-hydroxy-4-oxobut-2-enoate* (**6b**). Yield: 98%; mp: 151–153 °C. ^1^H-NMR (DMSO-*d*_6_): δ = 1.22 (t, *J* = 7.1, 3H, CH_3_), 2.23 (s, 3H, CH_3_), 3.82 (s, 3H, OCH_3_), 4.09 (q, *J* = 7.1, 2H, CH_2_), 5.32 (s, 2H, CH_2_), 6.54–7.64 (m, 9H, ArH and CH); Anal. calcd for C_23_H_23_NO_5_: C 70.21, H 5.89, N 3.56, found C 70.39, H 5.96, N 3.41.

*4-[1-(4-Methylbenzyl)-4-methoxy-1H-indol-3-yl]-2-hydroxy-4-oxobut-2-enoate* (**6c**). Yield: 99%; mp: 198 °C dec. ^1^H-NMR (DMSO-*d*_6_): δ = 1.22 (t, *J* = 7.1, 3H, CH_3_), 2.23 (s, 3H, CH_3_), 3.82 (s, 3H, OCH_3_), 4.12 (q, *J* = 7.1, 2H, CH_2_), 5.32 (s, 2H, CH_2_), 6.55–7.64 (m, 9H, ArH and CH); Anal. calcd for C_23_H_23_NO_5_: C 70.21, H 5.89, N 3.56, found C 70.09, H 6.02, N 3.41.

*4-[1-(2,3-Dimethylbenzyl)-4-methoxy-1H-indol-3-yl]-2-hydroxy-4-oxobut-2-enoate* (**6d**). Yield: 97%; mp: 172–174 °C. ^1^H-NMR (DMSO-*d*_6_): δ = 1.23 (t, *J* = 7.1, 3H, CH_3_), 2.11 (s, 3H, CH_3_), 2.24 (s, 3H, CH_3_), 3.85 (s, 3H, OCH_3_), 4.10 (q, *J* = 7.1, 2H, CH_2_) 5.36 (s, 2H, CH_2_), 6.53–7.40 (m, 8H, ArH and CH); Anal. calcd for C_24_H_25_NO_5_: C 70.75, H 6.18, N 3.44, found C 70.71, H 6.22, N 3.40.

4-[1-(2,4-Dimethylbenzyl)-4-methoxy-1*H*-indol-3-yl]-2-hydroxy-4-oxobut-2-enoate (**6e**). Yield: 98%; mp: 200–202 °C. ^1^H-NMR (DMSO-*d*_6_): δ = 1.25 (t, *J* = 7.1, 3H, CH_3_), 2.17 (s, 3H, CH_3_), 2.22 (s, 3H, CH_3_), 3.85 (s, 3H, OCH_3_), 4.10 (q, *J* = 7.1, 2H, CH_2_), 5.31 (s, 2H, CH_2_), 6.62–7.43 (m, 8H, ArH and CH); Anal. calcd for C_24_H_25_NO_5_: C 70.75, H 6.18, N 3.44, found C 70.58, H 6.34, N 3.28.

*4-[1-(2,5-Dimethylbenzyl)-4-methoxy-1H-indol-3-yl]-2-hydroxy-4-oxobut-2-enoate* (**6f**). Yield: 94%, mp: 180–182 °C. ^1^H-NMR (DMSO-*d*_6_): δ = 1.23 (t, *J* = 7.1, 3H, CH_3_), 2.15 (s, 6H, CH_3_), 3.85 (s, 3H, OCH_3_), 4.10 (q, *J* = 7.1, 2H, CH_2_), 5.30 (s, 2H, CH_2_), 6.64–7.41 (m, 8H, ArH and CH); Anal. calcd for C_24_H_25_NO_5_: C 70.75, H 6.18, N 3.44, found C 70.61, H 6.13, N 3.38.

*4-[1-(2,6-Dimethylbenzyl)-4-methoxy-1H-indol-3-yl]-2-hydroxy-4-oxobut-2-enoate* (**6g**). Yield: 91%, mp: 195–197 °C. ^1^H-NMR (DMSO-*d*_6_): δ = 1.18 (t, *J* = 7.1, 3H, CH_3_), 1.91 (s, 6H, CH_3_), 3.57 (s, 3H, OCH_3_), 4.02 (q, *J* = 6.8, 2H, CH_2_), 5.30 (s, 2H, CH_2_), 6.62–7.25 (m, 8H, ArH and CH); Anal. calcd for C_24_H_25_NO_5_: C 70.75, H 6.18, N 3.44, found C 70.82, H 6.27, N 3.31.

*4-[1-(3,5-Dimethylbenzyl)-4-methoxy-1H-indol-3-yl]-2-hydroxy-4-oxobut-2-enoate* (**6h**). Yield: 96%, mp: 270 °C dec. ^1^H-NMR (DMSO-*d*_6_): δ = 1.23 (t, *J* = 7.1, 3H, CH_3_), 2.18 (s, 6H, CH_3_), 3.82 (s, 3H, OCH_3_), 4.12 (q, *J* = 7.1, 2H, CH_2_), 5.28 (s, 2H, CH_2_), 6.58–7.65 (m, 8H, ArH and CH); Anal. calcd for C_24_H_25_NO_5_:C 70.75, H 6.18, N 3.44, found C 70.82, H 6.09, N 3.51.

*4-[1-(4-Fluoro-3,5-dimethylbenzyl)-4-methoxy-1H-indol-3-yl]-2-hydroxy-4-oxobut-2-enoate* (**6i**). Yield: 98%, mp 255–257 °C. ^1^H-NMR (DMSO-*d*_6_): δ = 1.22 (t, *J* = 7.1, 3H, CH_3_), 2.13 (s, 6H, CH_3_), 3.82 (s, 3H, OCH_3_), 4.09 (q, *J* = 7.1, 2H, CH_2_), 5.27 (s, 2H, CH_2_), 6.60–7.65 (m, 7H, ArH and CH); Anal. calcd for C_24_H_24_FNO_5_: C 67.75, H 5.69, N 3.29, found C 67.79, H 5.29, N 3.25.

#### 3.1.4. General procedure for the synthesis of 4-[1-benzyl-4-methoxy-1*H*-indol-3-yl]-2-hydroxy-4-oxobut-2-enoic acids **7a–i**

Following the synthetic procedure previously reported by us [17,18,20], the appropriate 4-[1-benzyl-4-methoxy-1*H*-indol-3-yl]-2-hydroxy-4-oxobut-2-enoate (1 mmol) was dissolved in methanol (5 mL) and treated with 2 N NaOH (5 mL, 50 mmol). The reaction mixture was stirred at room temperature for 1.5 h and then acidified with conc. HCl to give the corresponding hydrolyzed derivative. The desired products were crystallized from a mixture of ethanol and diethyl ether (1:4).

4*-[1-(2-Methylbenzyl)-4-methoxy-1H-indol-3-yl]-2-hydroxy-4-oxobut-2-enoic acid* (**7a**). Yield: 60%, mp 164–166 °C. ^1^H-NMR (DMSO-*d*_6_): δ = 2.30 (s, 3H, CH_3_), 3.92 (s, 3H, OCH_3_), 5.51 (s, 2H, CH_2_), 6.60–7.60 (m, 8H, ArH a\][0-9a-z]nd CH), 8.26 (s, 1H, ArH), 13.7 (bs, 1H, OH), 15.7 (bs, 1H, OH); ^13^C-NMR ([D_6_]DMSO): δ = 21.8, 56.2, 56.6, 103.4, 111.1, 111.7, 113.9, 121.1, 125.2, 125.7, 128.7, 134.8, 136.8, 152.1, 164.5, 187.6, 189.7 ppm; Anal. calcd for C_21_H_19_NO_5_: C 69.03, H 5.24, N 3.83, found C 69.07, H 5.28, N 3.87.

*4-[1-(3-Methylbenzyl)-4-methoxy-1H-indol-3-yl]-2-hydroxy-4-oxobut-2-enoic acid* (**7b**). Yield: 45%, mp 150–152 °C. ^1^H-NMR (DMSO-*d*_6_): δ = 2.24 (s, 3H, CH_3_), 3.89 (s, 3H, OCH_3_), 5.46 (s, 2H, CH_2_), 6.78–7.58 (m, 8H, ArH and CH), 8.50 (s, 1H, ArH); ^13^C-NMR ([D_6_]DMSO): δ = 24.6, 56.2, 59.4, 103.4, 111.1, 111.7, 113.9, 121.1, 125.2, 126.0, 128.6, 130.9, 134.8, 136.1, 137.5, 138.3, 152.1, 164.5, 187.6, 189.7 ppm; Anal. calcd for C_21_H_19_NO_5_: C 69.03, H 5.24, N 3.83, found C 69.11, H 5.17, N 3.94.

*4-[1-(4-Methylbenzyl)-4-methoxy-1H-indol-3-yl]-2-hydroxy-4-oxobut-2-enoic acid* (**7c**). Yield: 58%, mp 141–143 °C. ^1^H-NMR (DMSO-*d*_6_): δ = 2.24 (s, 3H, CH_3_), 3.89 (s, 3H, OCH_3_), 5.44 (s, 2H, CH_2_), 6.77–7.56 (m, 8H, ArH and CH), 8.48 (s, 1H, ArH); ^13^C-NMR ([D_6_]DMSO): δ = 24.3, 56.2, 59.1, 103.4, 111.1, 111.7, 113.9, 121.1, 125.2, 128.9, 133.2, 134.8, 135.4, 137.5, 152.1, 164.5, 187.6, 189.7 ppm; Anal. calcd for C_21_H_19_NO_5_: C 69.03, H 5.24, N 3.83, found C 68.97, H 5.11, N 3.73.

*4-[1-(2,3-Dimethylbenzyl)-4-methoxy-1H-indol-3-yl]-2-hydroxy-4-oxobut-2-enoic acid* (**7d**). Yield: 66%, mp 160–162 °C. ^1^H-NMR (DMSO-*d*_6_): δ = 2.18 (s, 3H, CH_3_), 2.26 (s, 3H, CH_3_), 3.91 (s, 3H, OCH_3_), 5.52 (s, 2H, CH_2_), 6.46–7.61 (m, 7H, ArH and CH), 8.20 (s, 1H, ArH); ^13^C-NMR ([D_6_]DMSO): δ = 19.3, 22.1, 56.2, 56.9, 103.4, 111.1, 111.7, 113.9, 121.1, 125.2, 125.6, 125.9, 134.8, 135.8, 136.7, 137.3, 137.5, 152.1, 164.5, 187.6, 189.7 ppm; Anal. calcd for C_22_H_21_NO_5_: C 69.65, H 5.58, N 3.69, found C 69.69, H 5.54, N 3.65.

*4-[1-(2,4-Dimethylbenzyl)-4-methoxy-1H-indol-3-yl]-2-hydroxy-4-oxobut-2-enoic acid* (**7e**). Yield: 60%, mp 143–145 °C. ^1^H-NMR (DMSO-*d*_6_): δ = 2.22 (s, 3H, CH_3_), 2.23 (s, 3H, CH_3_), 3.89 (s, 3H, OCH_3_), 5.42 (s, 2H, CH_2_), 6.58–7.93 (m, 7H, ArH e CH), 8.14 (s, 1H, ArH); ^13^C-NMR ([D_6_]DMSO): δ = 22.1, 24.6, 56.2, 56.6, 103.4, 111.1, 111.7, 113.9, 121.1, 125.2, 125.9, 128.8, 130.8, 134.4, 134.8, 135.3, 136.7, 137.5, 152.1, 164.5, 187.6, 189.7 ppm; Anal. calcd for C_22_H_21_NO_5_: C 69.65, H 5.58, N 3.69, found C 69.71, H 5.43, N 3.51.

*4-[1-(2,5-Dimethylbenzyl)-4-methoxy-1H-indol-3-yl]-2-hydroxy-4-oxobut-2-enoic acid* (**7f**). Yield: 59%, mp 163–165 °C. ^1^H-NMR (DMSO-*d*_6_): δ = 2.13 (s, 3H, CH_3_), 2.22 (s, 3H, CH_3_), 3.93 (s, 3H, OCH_3_), 5.46 (s, 2H, CH_2_), 6.56–7.63 (m, 7H, ArH e CH), 8.23 ppm (s, 1H, ArH); ^13^C-NMR (DMSO-*d*_6_): δ = 21.8, 24.6, 56.2, 56.6, 103.4, 111.1, 111.7, 113.9, 121.1, 125.2, 125.9, 128.8, 130.8, 133.8, 134.8, 135.3, 137.3, 137.5, 152.1, 164.5, 187.6, 189.7 ppm; Anal. calcd for C_22_H_21_NO_5_: C 69.65, H 5.58, N 3.69, found C 69.82, H 5.41, N 3.78.

*4-[1-(2,6-Dimethylbenzyl)-4-methoxy-1H-indol-3-yl]-2-hydroxy-4-oxobut-2-enoic acid* (**7g**). Yield: 76%, mp 173–175 °C. ^1^H-NMR (DMSO-*d*_6_): δ = 2.19 (s, 6H, CH_3_), 3.92 (s, 3H, OCH_3_), 5.40 (s, 2H, CH_2_), 6.85–7.44 (m, 7H, ArH e CH), 7.63 ppm (s, 1H, ArH); ^13^C-NMR (DMSO-*d*_6_): δ = 22.1, 54.1, 56.2, 103.4, 111.1, 111.7, 113.9, 121.1, 125.2, 125.6, 125.9, 134.8, 136.7, 137.5, 141.1, 152.1, 164.5, 187.6, 189.7 ppm; Anal. calcd for C_22_H_21_NO_5_: C 69.65, H 5.58, N 3.69, found C 69.49, H 5.72, N 3.48.

*4-[1-(3,5-Dimethylbenzyl)-4-methoxy-1H-indol-3-yl]-2-hydroxy-4-oxobut-2-enoic acid* (**7h**). Yield: 52%; mp: 120–122 °C. ^1^H-NMR (DMSO-*d*_6_): δ = 2.19 (s, 6H, CH_3_), 3.89 (s, 3H, OCH_3_), 5.41 (s, 2H, CH_2_), 6.78–7.59 (m, 7H, 6ArH and CH), 8.48 ppm (s, 1H, ArH); ^13^C-NMR DMSO-*d*_6_): δ = 24.1, 56.2, 59.7, 103.4, 111.1, 111.7, 113.9, 121.1, 125.2, 127.9, 134.8, 136.0, 137.5, 138.2, 152.1, 164.5, 187.6, 189.7 ppm; Anal. calcd for C_22_H_21_NO_5_: C 69.65, H 5.58, N 3.69, found: C 69.45, H 5. 41, N 3.75.

*4-[1-(4-Fluoro-3,5-dimethylbenzyl)-4-methoxy-1H-indol-3-yl]-2-hydroxy-4-oxobut-2-enoic acid* (**7i**). Yield: 40%; mp: 153–155 °C. ^1^H-NMR (DMSO-*d*_6_): δ = 2.15 (s, 6H, CH_3_), 3.89 (s, 3H, OCH_3_), 5.39 (s, 2H, CH_2_), 6.78–7.21 (m, 5H, ArH e CH), 7.57 (s, 1H, ArH), 8.47 ppm (s, 1H, ArH); ^13^C-NMR ([D_6_]DMSO): δ = 18.1, 56.2, 59.7, 103.4, 111.1, 111.7, 113.9, 121.1, 123.7, 125.2, 129.5, 131.6, 134.8, 137.5, 152.1, 160.5, 164.5, 187.6, 189.7 ppm; Anal. calcd for C_22_H_20_FNO_5_: C 66.49, H 5.07, N 3.52, found: C 66.36, H 5.28, N 3.39.

### 3.2. Biological Assays

*Overall integrase assay using an enzyme-linked immunosorbent assay (ELISA):* To determine the susceptibility of the HIV-1 integrase enzyme towards different compounds we used enzyme-linked immunosorbent assays. These assays use an oligonucleotide substrate of which one oligonucleotide (5’-ACTGCTAGAGATTTTCCACACTGACTAAAAGGGTC-3’) is labeled with biotin at the 3’ end and the other oligonucleotide is labeled with digoxigenin at the 5’ end. For the overall integration assay the second 5’-digoxigenin labeled oligonucleotide is (5’-GACCCTTTTAGTCAGTGTGGAAA ATCTCTAGCAGT-3’). For the Strand Transfer assay the second oligonucleotide lacks GT at the 3’ end. The integrase enzyme was diluted in 750 mM NaCl, 10 mM Tris pH 7.6, 10% glycerol, and 1 mM β-mercaptoethanol. To perform the reaction, diluted integrase (4 µL, corresponding to a concentration of 1.6 µM) and annealed oligonucleotides (4 µL, 7 nM) were added in a final reaction volume of 40 µL containing 10 mM MgCl_2_, 5 mM DTT, 20 mM HEPES pH 7.5, 5% PEG, and 15% DMSO. The reaction was carried out for 1 h at 37 °C. Reaction products were denatured with 30 mM NaOH and detected by an immunosorbent assay on avidin-coated plates [23].

*In vitro anti-HIV and drug susceptibility assays:* The inhibitory effect of antiviral drugs on the HIV-induced cytopathic effect (CPE) in human lymphocyte MT-4 cell culture was determined by the MT-4/MTT-assay [24]. This assay is based on the reduction of the yellow 3-(4,5-dimethylthiazol-2-yl)-2,5-diphenyltetrazolium bromide (MTT) by mitochondrial dehydrogenase of metabolically active cells to a blue formazan derivative, which can be measured spectrophotometrically. The 50% cell culture infective dose (CCID_50_) of the HIV(III_B_) strain was determined by titration of the virus stock using MT-4 cells. For the drug-susceptibility assays, MT-4 cells were infected with 100–300 CCID_50_ of the virus stock in the presence of fivefold serial dilutions of the antiviral drugs. The concentration of the various compounds that achieved 50% protection against the CPE of the different HIV strains, which is defined as the EC_50_, was determined. In parallel, the 50% cytotoxic concentration (CC_50_) was determined.

## 4. Conclusions

A novel series of indole derivatives was synthesized by means of an eco-friendly method which afforded the expected molecules quickly, in very high yields and using a very small amount of solvent. Although their biological data show that the modifications did not improve the biological profile of our lead compound “CHI-1043”, some HIV-1 INSTIs with an IC_50_ in the low micromolar range were obtained, thus affording further SAR information that can be considered useful for future studies.
